# An efficient watermarking algorithm for digital audio data in security applications

**DOI:** 10.1038/s41598-023-45619-w

**Published:** 2023-10-27

**Authors:** Mohamed Yamni, Achraf Daoui, Hicham Karmouni, Mhamed Sayyouri, Hassan Qjidaa, Saad motahhir, Ouazzani Jamil, Walid El-Shafai, Abeer D. Algarni, Naglaa F. Soliman, Moustafa H. Aly

**Affiliations:** 1 CED-ST, STIC, Laboratory of Electronic Signals and Systems of Information LESSI, Faculty of Science Dhar El Mahrez, University Sidi Mohamed Ben Abdellah, Fez, Morocco; 2 Engineering, Systems and Applications Laboratory, National School of Applied Sciences, Sidi Mohamed Ben Abdellah University, BP 72, My Abdallah Avenue Km. 5 Imouzzer Road, Fez, Morocco; 3 Systems and Sustainable Environment Laboratory (SED), Faculty of Engineering Sciences (FSI) Private, University of Fez (UPF), Fez, Morocco; 4 Security Engineering Lab, Computer Science Department, Prince Sultan University, 11586 Riyadh, Saudi Arabia; 5 Department Electronics and Electrical Communications Engineering, Faculty of Electronic Engineering, Menoufia University, Menouf, 32952 Egypt; 6 Department of Information Technology, College of Computer and Information Sciences, Princess Nourah bint Abdulrahman University, P.O. Box 84428, 11671 Riyadh, Saudi Arabia; 7 Electronics and Communications Engineering Department, College of Engineering and Technology, Arab Academy for Science, Technology and Maritime Transport, Alexandria, 1029 Egypt

**Keywords:** Electrical and electronic engineering, Mathematics and computing

## Abstract

Transform-domain audio watermarking systems are more robust than time-domain systems. However, the main weakness of these systems is their high computational cost, especially for long-duration audio signals. Therefore, they are not desirable for real-time security applications where speed is a critical factor. In this paper, we propose a fast watermarking system for audio signals operating in the hybrid transform domain formed by the fractional Charlier transform (FrCT) and the dual-tree complex wavelet transform (DTCWT). The central idea of the proposed algorithm is to parallelize the intensive and repetitive steps in the audio watermarking system and then implement them simultaneously on the available physical cores on an embedded systems cluster. In order to have a low power consumption and a low-cost cluster with a large number of physical cores, four Raspberry Pis 4B are used where the communication between them is ensured using the Message Passing Interface (MPI). The adopted Raspberry Pi cluster is also characterized by its portability and mobility, which are required in watermarking-based smart city applications. In addition to its resistance to any possible manipulation (intentional or unintentional), high payload capacity, and high imperceptibility, the proposed parallel system presents a temporal improvement of about 70%, 80%, and 90% using 4, 8, and 16 physical cores of the adopted cluster, respectively.

## Introduction

Recently, information security has received considerable attention due to the appearance of serious problems with multimedia data, such as illegal distribution, copying, authentication, and editing. Among the techniques followed to avoid these problems, digital watermarking is a technology used to ensure law enforcement and copyright protection of multimedia data. Digital watermarking methods secure multimedia data by embedding copyright information (known as a watermark) imperceptibly and securely into the host in a way that resists any possible manipulation, whether intentional or unintentional, that attempts to delete or damage the watermark.

Several watermarking methods for audio signal protection have been published in the literature, which can be mainly classified into two categories: time domain methods and transform domain methods. The methods in the first category^1, 2^ are the simplest; they embed the watermark by directly modifying the host signal samples. However, these methods are generally not very robust to various common signal processing manipulations. In contrast, the second category methods are more robust by embedding the watermark into transform coefficients; examples include the discrete wavelet transform (DWT)^3–5^, discrete cosine transform (DCT)^6^, singular value decomposition (SVD)^7^, and lifting wavelet transform (LWT)^8^. Moreover, to improve the performance, hybrid audio watermarking methods have been proposed that adopt two transforms, such as DWT-DCT^9^, DWT-DTMT^10^, and DWT-SVD^11^.

The main limitations of the existing audio watermarking systems are low robustness, in particular to shifting modification, and high computation cost, especially for long-duration audio.

Several methods address the first limitation that can be overcome by using a synchronization code strategy^12–18^. With this strategy, synchronization codes are also embedded together with a watermark into the host audio signal to determine the positions of the modified samples of the audio signal. In the watermark extraction process, these synchronization codes are firstly found, and then the watermark bits that follow the synchronization code can be extracted. Without using a synchronization code strategy, we proposed in^19^ a hybrid approach robust to attacks, including shifting attacks, based on the Dual Tree Complex Wavelet Transform (DTCWT) and the Fractional Charlier Transform (FrCT). We embedded the watermark in the host signal by manipulating the coefficients resulting from the application of the DTCWT and FrCT, respectively.

**Figure 1:**
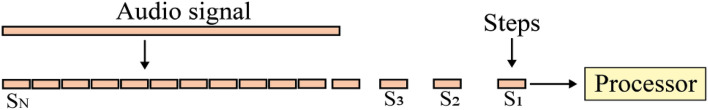
Sequential audio watermarking system.

If the robustness problem against shifting attacks has been effectively addressed by the methods mentioned earlier, the execution time of audio watermarking systems in real-time applications remains a challenging problem. In the context of copyright protection applications, the duration of the watermark embedding process may not be a primary concern, but the need for swift watermark extraction is of paramount importance^20^. This emphasis on rapid extraction is supported by a multitude of compelling reasons^21^. Firstly, in scenarios characterized by real-time content dissemination, such as live streaming or content delivery networks, the rapid extraction of watermarks becomes indispensable for immediate verification of authenticity and copyright ownership. Fast extraction is vital for detecting and addressing unauthorized usage or distribution promptly. Secondly, content creators and copyright holders frequently employ automated systems to monitor the utilization of their intellectual property across digital platforms. The efficient and timely tracking of copyrighted material depends on a swift watermark extraction process, facilitating the effective implementation of enforcement measures. Thirdly, the user experience is significantly affected by the pace of watermark extraction, particularly in applications like video streaming or online gaming. The imperative here is to minimize disruptions and latency issues, ensuring seamless content consumption. Fourthly, scalability considerations loom large as the volume of multimedia content burgeons across the internet. A rapid watermark extraction capability is pivotal for the efficient management and safeguarding of extensive content repositories. Fifthly, the expeditious extraction of watermarks plays a crucial role in deterring piracy and curtailing unauthorized distribution of copyrighted content. It strengthens the ability to promptly identify infringements and take necessary legal actions, thereby effectively safeguarding intellectual property rights. These reasons underscore the significance of fast watermark extraction in the context of audio watermarking systems used for copyright protection. However, most audio watermarking systems in the transform domain, such as^3, 9–11, 19^, are very time-consuming, especially for signals of long duration. These systems operate in a sequential manner (Fig. 1). They divide the audio signal into segments and then apply a set of steps to each segment (preprocessing, switching from the time domain to the transform domain, embedding watermark bits, reconstructing watermarked segments, etc.). Only one segment is processed at a time on a single processor core. Applying transforms, and inverse transforms in a sequential way on audio segments are intensive processes, mainly for audio signals of long duration and for hybrid approaches that combine multiple transforms.

**Figure 2:**
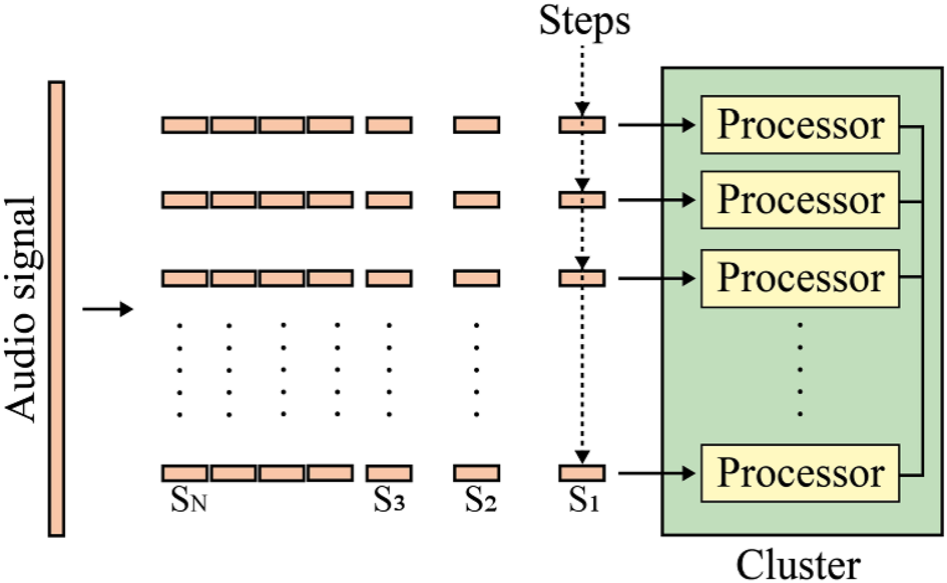
Proposed parallel audio watermarking system.

The main goal of this paper is to create and implement a fast audio watermarking system in the transform domain that can be executed in real-time. The central idea of the possible solution is to parallelize the intensive and repetitive steps in the audio watermarking system and then execute them simultaneously on the available physical cores of a multi-core processor (Fig. 2).

In the realm of parallel computing, various endeavors have been made, particularly in the domain of image processing applications. For instance, Hosny et al.^22^ presented a pioneering parallel medical image watermarking scheme, which they successfully deployed on both multi-core CPUs and GPUs. Similarly, Daoui et al.^23^ introduced a parallel image encryption algorithm tailored for multi-core CPU architectures. Additionally, researchers in a related study^24^ harnessed the parallel processing capabilities of both multi-core CPUs and GPUs to enhance image reconstruction and image classification tasks.

Despite the documented strides made in leveraging parallel computing for computational acceleration in various domains, these efforts have predominantly been confined to conventional personal computing devices (desktops or laptops). Such devices, distinguished by their considerable physical dimensions and weight, possess limited portability. Consequently, their applicability in mobility-constrained environments, encompassing scenarios like transportation modes (e.g., cars, trains, planes, and boats) and smart home or urban infrastructure contexts, has remained largely impractical.

In response to these inherent limitations associated with traditional personal computing systems, the adoption of mobile and portable embedded systems, exemplified by platforms such as Raspberry Pis, has emerged as a viable solution^25^. These embedded systems offer a compelling alternative by virtue of their compact form factor, lower power consumption, and enhanced mobility, making them well-suited for a diverse range of applications and settings.

Parallel processing entails a heightened demand for computational resources, encompassing processor cores and memory, due to the concurrent execution of tasks and the need for efficient workload distribution. Simultaneous execution of multiple tasks necessitates the allocation of dedicated processor cores, while data sharing and synchronization among these tasks amplify the requirement for memory resources. To tackle this computational challenge, we build in this paper a cluster based on several Raspberry Pis for fast, parallel, and distributed audio watermarking. The selection of the Raspberry Pi as our computational platform is substantiated by its advantageous features, including its exceptional portability due to its lightweight (46 g) and compact dimensions (85.6 mm × 56.5 mm), coupled with its minimal power consumption and affordability. Compared to other versions of the Raspberry Pi, the 4B version with 2 GB of RAM is powerful enough to support complex signal processing applications that require a high computational load. In this paper, a cluster based on four Raspberry Pis 4B is built to have a large number of physical cores, which is very useful to accelerate the time of a parallel watermarking system.

This paper presents a parallel watermarking system for audio signals, implemented on the Raspberry Pi cluster. The proposed approach decomposes the host audio signal and the watermark into several sub-signals and vectors equal to the number of available cores of the Raspberry Pi cluster. Then simultaneously, on each core, we extract from each sub-signal the low-frequency coefficients that are less sensitive to the human auditory system by applying the 5-level DTCWT. Then, we apply the FrCT transform^26^ with the optimal fractional order in order to improve the imperceptibility and robustness, and then we embed the watermark bits by quantizing the energies of FrCT coefficients. Finally, each core of the Raspberry Pi cluster sends the watermarked sub-signal to the master Raspberry Pi, and then the latter combines all these sub-signals to obtain the watermarked audio signal. Each Raspberry Pi 4B in our cluster has the same input data and the same copy of the instruction script, but each Raspberry Pi executes only a specific part of the script determined by the master Raspberry Pi of the cluster. Raspberry Pis in the cluster are independent of each other, and communications (sending and receiving data) between them are ensured using the Message Passing Interface (MPI) library^27^.

Like the embedding process, the watermark extraction process requires neither the original audio signal nor the original watermark (blind extraction). We also used a modified Henon map^28^ to encrypt the watermark and guarantee security.

The results show that the proposed parallel watermarking system is fast compared to the sequential system, with an improvement of about 70%, 80%, and 90% using 4, 8, and 16 cores of the Raspberry Pi cluster, respectively.

As summary, the contributions of this article are presented as follows.

- A new parallel audio watermarking system implemented on the embedded systems cluster is proposed for the first time.
- The audio watermarking system is fast and can be desirable for real-time applications.
- All the Raspberry Pis in the cluster work simultaneously on the audio watermarking system, which reduces the execution time.
- Raspberry Pi is characterized by its easy portability due to its lightweight and small size, and therefore, the limited portability of standard PCs can be overcome.

The rest of the manuscript is organized as follows: Sections "Discrete fractional Charlier transform", "Dual-tree complex wavelet transform", and "Modified Henon map" present respectively the FrCT, the DTCWT, the modified Henon map, and their roles in the proposed approach. Section "Raspberry Pi cluster" presents our Raspberry Pi cluster. Section "Proposed parallel audio watermarking system" presents the proposed parallel audio watermarking system. Section "Experiments results" presents the experimental results and discussions, and the conclusion is finally provided in Section "Conclusion".

## Discrete fractional Charlier transform

In our previous paper^26^, we proposed the fractional version of the Charlier transform, which is called the fractional Charlier transform (FrCT) based on the fractional Charlier polynomials (FrCPs) also proposed in the same paper. The FrCT generalizes the classical Charlier transform of integer order to fractional order in order to benefit the properties of non-integer orders.

The main property of FrCT that makes it very suitable for digital watermarking is its dependence on transform orders. By adjusting the fractional orders in the FrCT transform, different FrCT coefficients can be obtained. Therefore, we select the optimal fractional orders, and the corresponding FrCT coefficients are used as host coefficients to integrate the watermark. This approach improves the imperceptibility and robustness requirements of the watermarking system. In addition, the fractional orders in the transform can be used as additional secret keys to improve the security of the watermarking system.

Let $x(t), \, t = 1,2,...,N$ be a one-dimensional signal of finite length $N$, the one-dimensional fractional Charlier transform of this signal with fractional order $\alpha , \, (\alpha \in R)$ is defined as follows:

1 $${\varvec{FrCM}}^{\alpha } = {\varvec{C}}^{\alpha } {\varvec{x}}$$

where ${\varvec{x}}$ is a column vector representation of $x(t)$, and ${\varvec{C}}^{\alpha }$ is the fractional Charlier polynomial matrix of size $N \times N$ and fractional order $\alpha$, which is defined as follows:

2 $${\varvec{FrCM}}^{\alpha } = {\varvec{C}}^{\alpha } {\varvec{x}}$$

where the eigenvectors of the fractional Charlier polynomial matrix $v_{k} (k = 0,1,...,N - 1)$ are the *k*th column of ${\mathbf{V}}$, and ${\mathbf{D}}^{\alpha }$ is defined as follows:

3 $${\mathbf{D}}^{\alpha } = Diag\{ 1,e^{ - j\alpha \pi } ,e^{ - j2\alpha \pi } , \ldots ,e^{ - j(N - 1)\alpha \pi } \}$$

The corresponding inverse transform (iFrCT) can be written as follows:

4 $${\varvec{x}} = {\varvec{C}}^{ - \alpha } {\varvec{FrCM}}^{\alpha }$$

## Dual-tree complex wavelet transform

**Figure 3:**
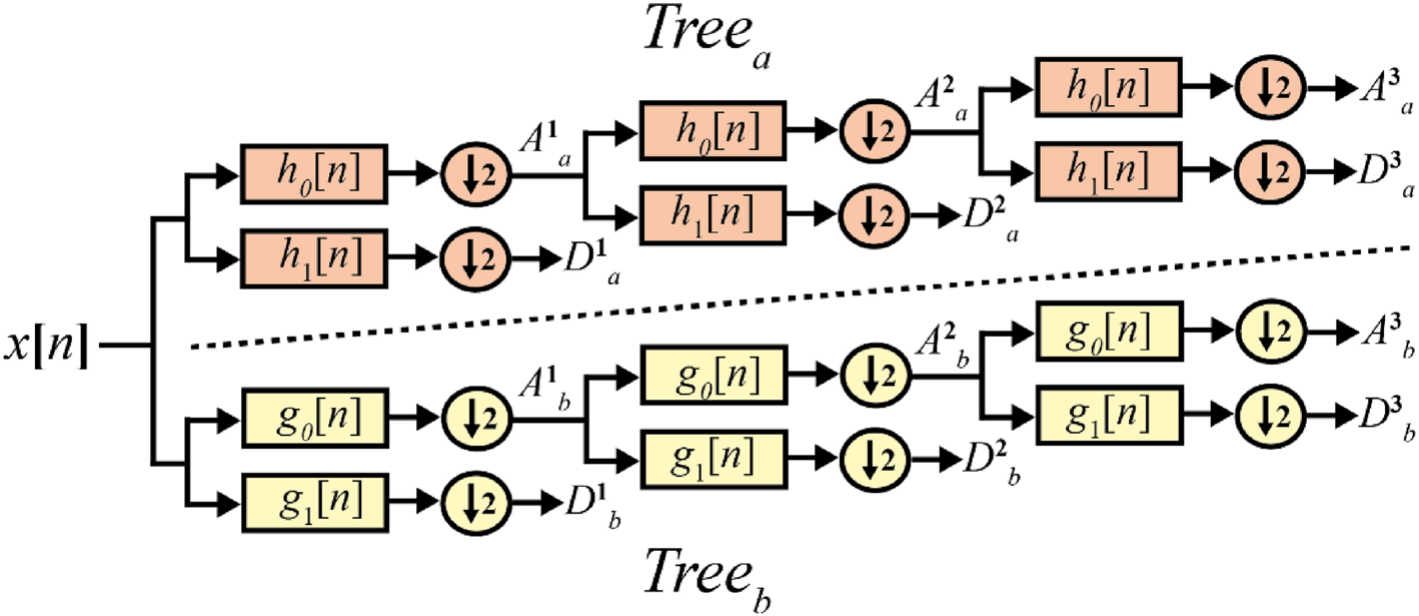
3-level DTCWT decomposition.

The DTCWT^29^ is an enhanced expansive version of the DWT. It is implemented as two separate DWTs ($Tree_{a}$ and $Tree_{b}$) applied on the same signal data (Fig. 3). At the heart of DTCWT is a pair of filters: low pass and high pass. For a DTCWT of level $H$, the low-pass ($h_{0}$) and high-pass ($h_{1}$) filters of $Tree_{a}$ generating the approximation coefficients $A_{a}^{H}$ (low frequencies) and the detail coefficients $D_{a}^{H} ,D_{a}^{H - 1} ,...,D_{a}^{1}$ (high frequencies). Similarly, the approximation coefficients $A_{b}^{H}$ and the detail coefficients $D_{b}^{H} ,D_{b}^{H - 1} ,....,D_{b}^{1}$ are generated by the low-pass and high-pass filters of $Tree_{b}$ $\{ g_{0} ,g_{1} \}$.

The outputs of the DTCWT can be interpreted as complex coefficients as follows:

5 $$A^{H} = A_{a}^{H} + jA_{b}^{H} {\text{ and }}D^{H} = D_{a}^{H} + jD_{b}^{H}$$

where $A^{H}$ are the approximation coefficients of level $H$ and $D^{H}$ are the detail coefficients of level $H$.

The original signal can be reconstructed without loss of information using inverse DTCWT (iDTCWT)^29^.

The main advantage of DTCWT for signal processing is the shifting invariance that is not ensured by DWT. Indeed, the DTCWT is approximately shifting invariant, which means that small shifts in the input signal do not produce major variations in the energy distribution of the DTCWT coefficients at different levels. To obtain this advantage, the approximation and the detail coefficients of $Tree_{a}$ must be approximate Hilbert transforms of the approximation and the detail coefficients of $Tree_{b}$, that is

6 $$A_{a}^{H} = {\varvec{\mathcal{H}}}(A_{b}^{H} ){\text{ and }}D_{a}^{H} = {\varvec{\mathcal{H}}}(D_{b}^{H} ) \,$$

where $\varvec{\mathcal{H}}$ is the Hilbert transform operator.

In our case, for the first level, we use a set of filters from^30^, and for the other levels, we use a set of filters from^31, 32^ in order to verify the condition of Eq. (6).

### Ethics approval

The manuscript does not contain any human or animal studies.

### Consent to participate

All authors are contributing and accepting to submit the current work.

## Modified Henon map

The modified Henon map is a nonlinear chaotic map very sensitive to the initial conditions recently proposed in^28^. This chaotic map is defined as follows:

7 $$\left\{ \begin{gathered} y(i) = b\left( {1 - d\left| {\sin \left( {y(i - 1)} \right)} \right|} \right)x(i - 1) \hfill \\ x(i) = 1 - a\left( {1 - c\left| {cos(i)} \right|} \right)x^{2} (i - 1) + y(i - 1) \hfill \\ \end{gathered} \right.;{\text{ with }}i = 0,1,2,...$$

where $$a \in [0.54,2], \, b \in [0,1], \, c \in [0,0.8],{\text{ and }}d \in [0,0.8][0,1]$$$$, \, c \in [0,0.8],{\text{ and }}d \in [0,0.8]$$ are the control parameters of the chaotic system. If $$c = d = 0$$, the modified Henon map coincides with the classical Henon map^33^:

8 $$\left\{ \begin{gathered} y(i) = bx(i - 1) \hfill \\ x(i) = 1 - ax^{2} (i - 1) + y(i - 1) \hfill \\ \end{gathered} \right.;{\text{ with }}i = 0,1,2,...$$

In this paper, the modified Henon map is used to encrypt the watermark information before embedding it into the original host audio signal. This makes the watermark hard to be extract by unauthorized persons, which improves the overall security of the audio watermarking system. In addition, the encryption of the watermark eliminates the correlation between its information, and consequently, an improvement can be achieved in terms of the overall robustness of the proposed watermarking system.

Let $W = \{ w(i), \, 0 \le i < N\}$ be a binary sequence of ones and zeros with *N* bits, the watermark encryption process is as follows:

(1) Generate a chaotic sequence ${\text{Y}} = \{ {\text{y(i)}},\,\,\,0 \le {\text{i}} < {\text{N}}\}$ using the modified Henon map (Eq. 7).

(2) Binarize the sequence ${\text{Y}}$ using its mean ${\text{T}}$ as a binarization threshold as follow:

9 $$\overline{{\text{Y}}} {\text{(i)}} = \left\{ \begin{gathered} {\text{1, if }}\,{\text{Y(i)}} \ge {\text{T}} \hfill \\ {\text{0, if }}\,{\text{Y(i)}} < {\text{T}} \hfill \\ \end{gathered} \right.,{ (0} \le {\text{i}} < {\text{N)}}$$

(3) Encrypt the watermark from ${\text{W}}$ to ${\text{W}}_{1}$ by applying the xor operation between ${\text{W}}$ and $\overline{{\text{Y}}}$ as follows:

10 $${\text{W}}_{1} = {\text{xor(W}},\overline{{\text{Y}}} )$$

The watermark can be decrypted by applying the xor operation between the encrypted watermark ${\text{W}}_{1}$ and the chaotic sequence $\overline{{\text{Y}}}$ as follows:

11 $${\text{W}} = {\text{xor(W}}_{1} ,\overline{{\text{Y}}} {)}$$

Watermark decryption depends on the initial parameters of the modified Henon map $\left\{ {a,b,c,d,x_{0} ,y_{0} } \right\}$. These parameters can be used as a secret key in an audio watermarking system.

## Raspberry Pi cluster

Basically, a cluster can be considered as a group of computers in a single entity. By combining two or more computers in a cluster, one can achieve a potential increase in performance by performing operations in a distributed and parallel environment. In this paper, we build a cluster using Raspberry Pi embedded systems for fast, parallel, and distributed audio watermarking. This choice can be justified by the fact that the Raspberry Pi is characterized by its easy portability due to its light weight (46 g) and small size (85.6 mm × 56.5 mm), low power consumption, low cost, and in terms of its functionality and scalability. Raspberry Pi has been used in various domains such as Internet of Things (IoT)^34–36^, image processing^37–40^, home automation^41, 42^, and other applications.

**Figure 4:**
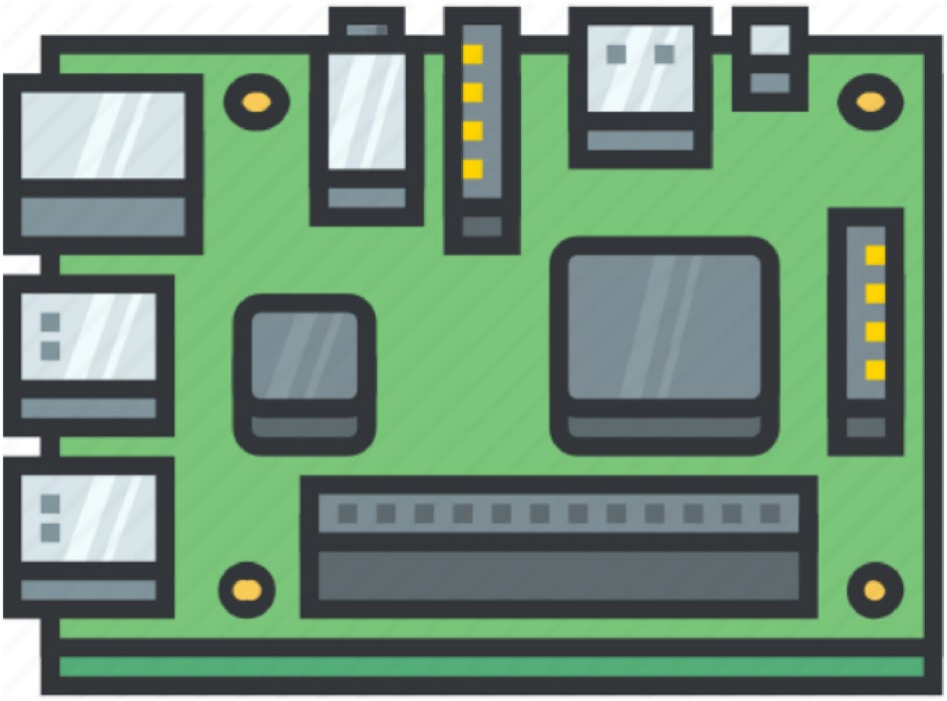
Raspberry Pi 4B computer.

Several versions of the Raspberry Pi computer have been produced by the Raspberry Pi Foundation^43^ with an open-source platform. Compared to the previous versions of the Raspberry Pi (3B, 3B + , 2B, 2B + , 1A, and 1B), Raspberry Pi 4B (Fig. 4) presents a major improvement in terms of processor speed and RAM quantity. The characteristics of the Raspberry Pi 4B are summarized in Table 1. As can be seen in Table 1, the Raspberry Pi 4B is powerful enough to support complex signal processing applications that require a high computational load. In addition, the Raspberry Pi 4B's processor has four physical cores, so it can be very useful when applications implemented on this processor can be run on more than one core. In this paper, a cluster based on four Raspberry Pis 4B is built to have a large number of processor cores, which is very useful to accelerate the time of an audio watermarking system.

**Table 1:** Raspberry Pi 4B characteristics.

Feature	Description
Soc	Broadcom BCM2711
Processor	Quad-core Cortex-A72 (ARM v8) 64-bit @ 1.5 GHz
RAM	2 GB
SD card support	Micro SD card slot for loading operating system and data storage
Connectivity	2.4 GHz and 5.0 GHz IEEE 802.11b/g/n/ac wireless LAN, Bluetooth 5.0, BLE Gigabit Ethernet 2 × USB 3.0 ports 2 × USB 2.0 ports
GPIO	Standard 40-pin GPIO header
Video and sound	2 × micro HDMI ports (up to 4Kp60 supported) 2-lane MIPI DSI display port 2-lane MIPI CSI camera port 4-pole stereo audio and composite video port
Input power	5 V via USB-C connector or GPIO header (minimum 3A)

**Figure 5:**
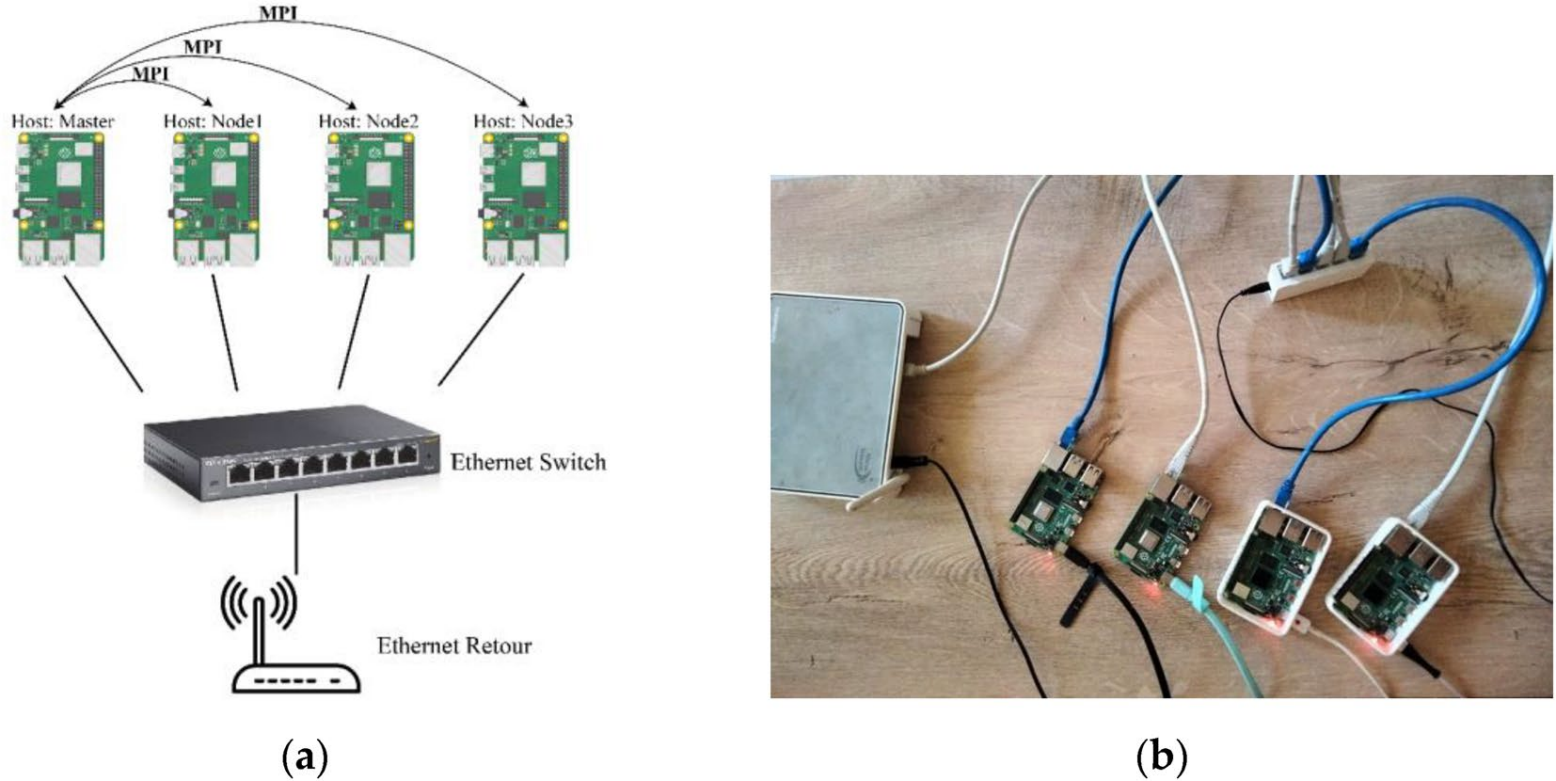
(**a**) Architecture of our Raspberry Pi cluster; (**b**) close-up of our Raspberry Pi cluster.

Figure 5 shows the architecture of our Raspberry Pi cluster: we have the main node (Master) that controls all operations and three computing nodes (Node1, Node2, Node3) to increase overall performance. Each node is equipped with 2 GB of RAM and a 16 GB SD card for local storage. The four Raspberry Pis were connected to an Ethernet router through the Ethernet switch.

In order to ensure communications between the four Raspberry Pis, we mainly need the MPICH tool. MPICH with Python wrapper (MPI4PY) is an open-source implementation of the MPI standard (Message Passing Interface)^27^, whose purpose is to manage parallel computer architectures. MPI allows the main Raspberry Pi (master node) to distribute, in a parallel manner, the computational task among all the other Raspberry Pis in the cluster.

After installing the same Raspbian OS and the same applications and libraries on all the Raspberry Pis, we configure their hostnames, and then we get their IP addresses. Finally, we authorize the master Raspberry Pi to connect to the other Raspberry Pis via SSH (Secure Shell) without a password.

**Figure 6:**
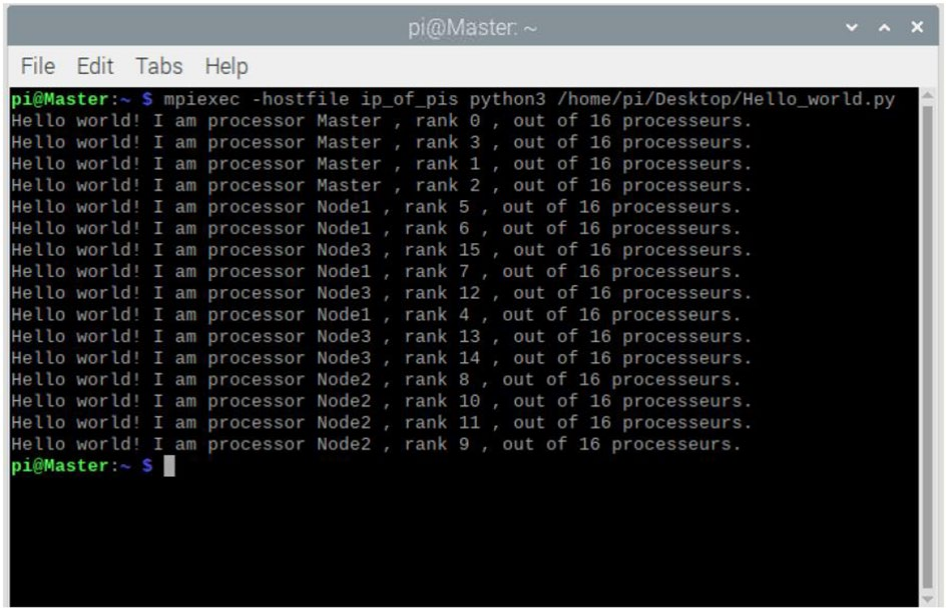
Basic functionality testing of our cluster.

Figure 6 shows the execution result of a simple Python script sent by the master Raspberry Pi to the other Raspberry Pis using MPI. Each of the 16 processors on the network had to report to the master to confirm that all processors were working properly.

It is essential to highlight that the master node may occasionally undergo an automatic restart when handling computationally-intensive tasks, especially if the power source is inadequate. To mitigate this issue, we employed power sources capable of delivering a stable current range of 2.0 to 2.5 amperes to each Raspberry Pi in our cluster.

The choice to opt for cluster computing in this paper over other computing techniques, such as cloud computing, is a strategic choice rooted in several key considerations. Firstly, data privacy and security are paramount in our audio watermarking system. Cluster computing allows us to maintain full control over our data, keeping it within our network. This level of control mitigates potential risks associated with relying on cloud-based storage and processing, where data may be exposed to external vulnerabilities. Furthermore, the nature of audio watermarking demands low-latency communication to ensure real-time processing. Cluster computing excels in this regard as it involves physically proximate nodes, reducing communication latency significantly compared to the internet-based data transfer typical of cloud computing. This low-latency advantage is critical for the timely execution of audio watermarking tasks. Additionally, audio watermarking is a computationally intensive process that requires tailored hardware and software configurations for optimal performance. Cluster computing offers us the flexibility to fine-tune these configurations to specifically meet the demands of our task. In contrast, cloud computing often involves shared resources, making it less customizable and potentially less efficient for our resource-intensive processing needs. Lastly, in terms of long-term cost-efficiency, cluster computing emerges as the preferred choice. Unlike cloud computing, which often incurs recurring service fees, cluster computing allows us to leverage our existing hardware investments without incurring ongoing expenses. This cost-saving aspect aligns well with our project's budgetary constraints.

## Proposed parallel audio watermarking system

**Figure 7:**
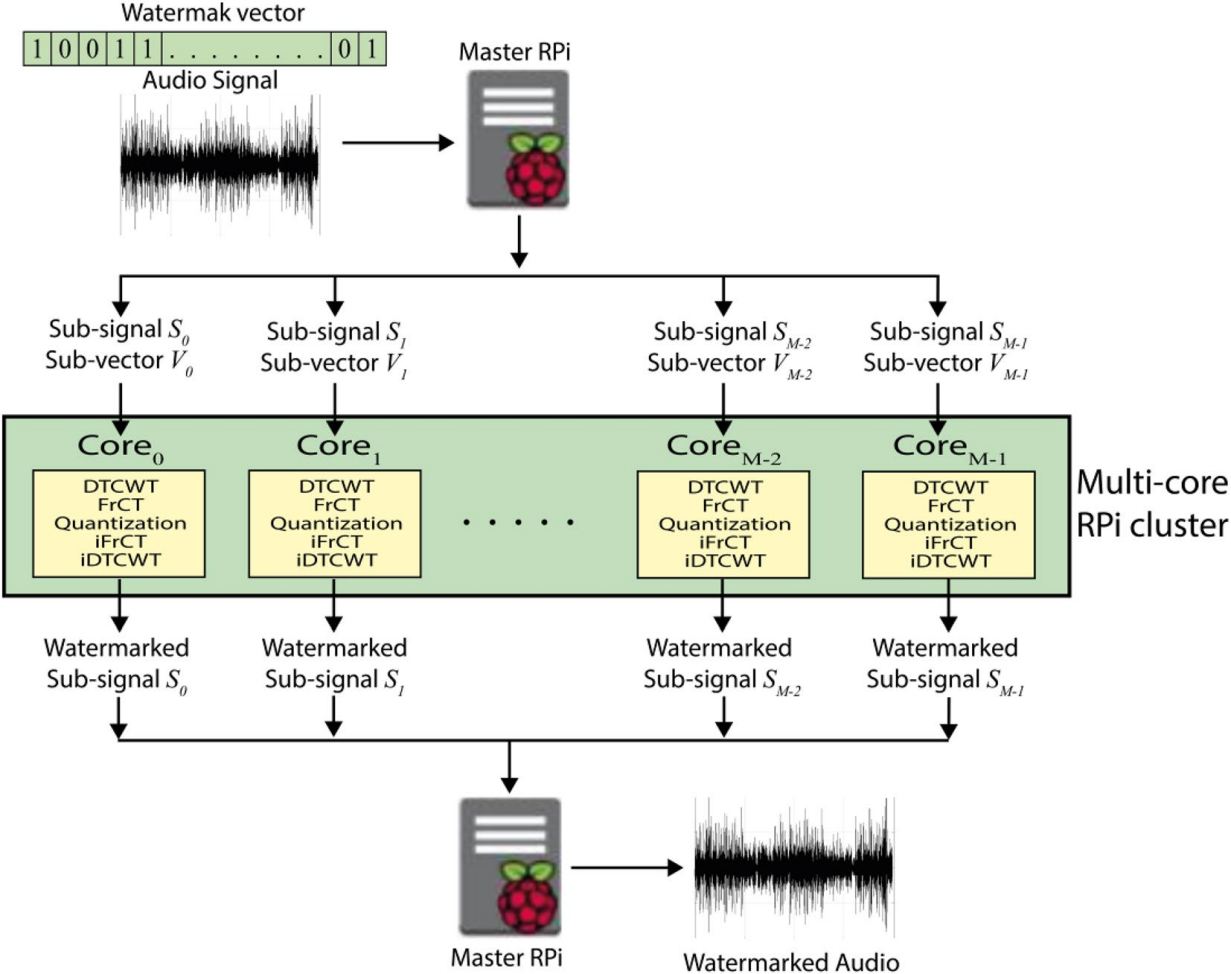
The flowchart of the proposed parallel audio watermarking scheme implemented on RPi cluster.

In order to accelerate the execution time, we propose a parallel audio watermarking system that can be implemented on the Raspberry Pi cluster. The proposed approach (Fig. 7) decomposes the host audio signal and the watermark into several sub-signals and vectors equal to the number of available cores of the Raspberry Pi cluster. Then, simultaneously on each core, we extract from each sub-signal the low-frequency coefficients by applying the 5-level DTCWT. Then, we apply the FrCT transform with the optimal fractional order in order to improve the imperceptibility and robustness, and then we embed the watermark bits by quantizing the energies of the first coefficients. Finally, each core of the Raspberry Pi cluster sends the watermarked sub-signal to the master node, and then the latter combines all these sub-signals to obtain the watermarked audio signal.

The watermark extraction process in the proposed system neither needs the original audio signal nor the original watermark (the extraction is blind). The watermarked audio signal is decomposed into sub-signals, and each sub-signal is sent to a single core of the Raspberry Pi cluster. Then, each sub-signal is subjected again to DTCWT and FrCT transforms before extracting the watermark bits. Finally, each core in the Raspberry Pi cluster sends the watermark bits to the master node, and then the latter node combines these bits to recover the watermark.

Each Raspberry Pi in our cluster has the same input data and the same instruction script, but each Raspberry Pi executes only a specific part of the script determined by the master Raspberry Pi of the cluster. All Raspberry Pis in the cluster are independent of each other, and communications (sending and receiving data) between the master Raspberry Pi and the other Raspberry Pi are ensured using the MPI library.

The following sections detail the embedding and extraction processes.

### Embedding process

Let $S = \{ s(i), \, 0 \le i < L\}$ denote a host audio signal with $L$ samples, and $W = \{ w(i) \in \{ 0,1\} , \, 0 \le i < N\}$ is a binary sequence of ones and zeros with $N$ bits to be embedded within the host audio signal. The watermark embedding process can be summarized as follows.

**Step 1:** Encrypt the watermark from $W$ to $W_{1}$ using the modified Henon map-based encryption procedure (Section "Modified Henon map") where the encrypted watermark is defined as follows:

12 $$W_{1} = \{ w_{1} (i) \in \{ 0,1\} , \, 0 \le i < N\}$$

The initial parameters of the modified Henon map $\left\{ {a,b,c,d,x_{0} ,y_{0} } \right\}$ labelled as $KEY$ are used as a secret key in our audio watermarking system.

**Step 2:** Divide $W_{1}$ into *M*-equal-length vectors $V_{k}$, where

13 $$V_{k} = \{ v_{k} (j), \, 0 \le j < J, \, J = N/M, \, k = 0,1,...,M - 1 \}$$

**Step 3:** Divide the audio signal $S$ into *M*-equal-length sub-signals $S_{k}$, where

14 $$S_{k} = \{ s_{k} (i), \, 0 \le i < L/M, \, k = 0,1,2...,M - 1\}$$

where *M* represents the number of available cores in the Raspberry Pi cluster.

Each core of the Raspberry Pi cluster $(core_{k} ,k = 0,1,...,M - 1)$ receives the watermark vector $V_{k}$ and sub-signal $S_{k}$ and then executes the steps (4–11).

**Step 4:** Decompose the sub-signal $S_{k}$ into $\, J$ frames, where $\, J = N/M$.

For each frame ($F_{j} ,j = 0,1,...,J$) apply the steps (5–10).

**Step 5:** Generate $A^{5}$ and $D^{5} ,D^{4} ,D^{3} ,D^{2} ,D^{1}$ by applying 5-level DTCWT, where $A^{5} = A_{a}^{5} + jA_{b}^{5}$ are the approximation coefficients of level 5 and $D^{i} = D_{a}^{i} + jD_{b}^{i} , \, i = 1,2,3,4,5$ are the detail coefficients of level $i$.

**Step 6:** Apply FrCT on $A^{5}$ produces vector named ${\varvec{FrCM}}^{\alpha }$

15 $${\varvec{FrCM}}^{\alpha } = {\varvec{C}}^{\alpha } {\varvec{A}}^{5}$$

where ${\varvec{FrCM}}^{\alpha }$ and ${\varvec{A}}^{5}$ are $1 \times n$ vectors, $n = \frac{L}{{N \times 2^{5} }}$, and ${\varvec{C}}^{\alpha }$ is the $n \times n$ matrix FrCPs which can be calculated from Eq. (2). In this paper, the fractional order in FrCT is set to $\alpha = 0.2$ as recommended in^19^.

**Step 7:** Calculate the energy of ${\varvec{FrCM}}^{\alpha }$ produced value named $E$.

**Step 8:** Embed the watermark bit in the vector ${\varvec{FrCM}}^{\alpha }$ by quantizing its energy $E$, in the following way

16 $$\overline{{{\varvec{FrCM}}^{\alpha } }} = E \times {\varvec{FrCM}}^{\alpha } /\overline{E}$$

where ${\varvec{FrCM}}^{\alpha }$ is the original FrCT vector, $\overline{{{\varvec{FrCM}}^{\alpha } }}$ is watermarked FrCT vector and

17 $$\, \overline{E} = \left\{ \begin{gathered} floor\left( {{E \mathord{\left/ {\vphantom {E \Delta }} \right. \kern-0pt} \Delta }} \right) + {{3\Delta } \mathord{\left/ {\vphantom {{3\Delta } 4}} \right. \kern-0pt} 4}{\text{ if }}v_{k} (j) = 1 \hfill \\ floor\left( {{E \mathord{\left/ {\vphantom {E \Delta }} \right. \kern-0pt} \Delta }} \right) + {\Delta \mathord{\left/ {\vphantom {\Delta 4}} \right. \kern-0pt} 4}{\text{ if }}v_{k} (j) = 0 \hfill \\ \end{gathered} \right.$$

where Δ is the quantization step and $floor(.)$ is the floor operator.

**Step 9:** Apply iFrCT on the watermarked vector $\overline{{{\varvec{FrCM}}^{\alpha } }}$ and obtain the watermarked approximation coefficients $\overline{{{\varvec{A}}^{5} }}$

18 $$\overline{{{\varvec{A}}^{5} }} = {\varvec{C}}^{ - \alpha } \overline{{{\varvec{FrCM}}^{\alpha } }}$$

**Step 10:** Get watermarked frame $\overline{{F_{j} }}$ by applying iDTCWT on $\overline{{{\varvec{A}}^{5} }}$ and $D^{5} ,D^{4} ,D^{3} ,D^{2} ,D^{1}$.

**Step 11:** Reconstruct the watermarked sub-signal $\overline{{S_{k} }}$ with watermarked frames:

19 $$\overline{{S_{k} }} = \{ \overline{{F_{j} }} , \, j = 0,1,...,J\}$$

**Step 12:** Each core of the cluster $(core_{k} , \, k = 0,1,...,M - 1)$ sends the watermarked sub-signal $\overline{{S_{k} }}$ to the $core_{0}$, and then the latter combines all these sub-signals to obtain the watermarked audio signal $\overline{S}$ as follows:

20 $$\overline{S} = \{ \overline{{S_{k} }} , \, k = 0,1,...,M - 1\}$$

### Extraction process

Let $\overline{S} = \{ \overline{s(i)} , \, 0 \le i < L\}$ denote a watermarked audio signal with $L$ samples, the extraction of the watermark from $\overline{S}$ is blind, and it can be summarized as follows:

**Step 1:** Divide the watermarked audio signal $\overline{S}$ into *M*-equal-length sub-signals $\overline{{S_{k} }}$, where *M* represents the number of Raspberry Pi cluster cores.

Each core of the Raspberry Pi cluster $(core_{k} , \, k = 0,1,...,M - 1)$ receives the sub-signal $\overline{{S_{k} }}$ and then executes the following steps.

**Step 2:** Decomposed the sub-signal $\overline{{S_{k} }}$ into $\, J$-equal-length frames, where $\, J = N/M$.

**Step 3:** For each frame ($\overline{{F_{j} }} ,0 \le j < J$), apply the steps **(5 ~ 7)** of the embedding process to obtain energy $\overline{E}$, then, apply the following extraction rule:

21 $$v_{k}^{*} (j) = \left\{ \begin{gathered} 1,{\text{ if }}\overline{E} - floor\left( {{{\overline{E} } \mathord{\left/ {\vphantom {{\overline{E} } \Delta }} \right. \kern-0pt} \Delta }} \right) \times \Delta \ge \frac{\Delta }{2} \, \hfill \\ 0,{\text{ if }}\overline{E} - floor\left( {{{\overline{E} } \mathord{\left/ {\vphantom {{\overline{E} } \Delta }} \right. \kern-0pt} \Delta }} \right) \times \Delta < \frac{\Delta }{2} \, \hfill \\ \end{gathered} \right.$$

where $V_{k}^{*} = \{ v_{k}^{*} (j), \, 0 \le j < J, \, k = 0,1,...,M - 1\} \,$ are the extracted encrypted watermark vectors, and Δ is the quantization step size.

**Step 4:** Each core in the Raspberry Pi cluster ($core_{k}$, where $k = 0,1...,M - 1$) sends its vector, $V_{k}^{*}$, to $core_{0}$, which then combines these vectors to obtain the encrypted watermark sequence as follows:

22 $$W_{1}^{*} = \{ V_{k}^{*} , \, 0 \le k < M\} {\text{ where }}V_{k}^{*} = \{ v_{k}^{*} (j),0 \le j < J, \, J = N/M\}$$

**Step 5:** $W_{1}^{*}$ is decrypted using the same initial parameters ($KEY$) of the modified Henon map to recover the watermark $W^{*}$.

## Experiments results

The performance of the proposed parallel audio watermarking system is demonstrated using the Python programming language. The proposed system is implemented on the Raspberry Pi cluster presented in Section "Raspberry Pi cluster", which is composed of four Raspberry Pi 4Bs, each equipped with 2 GB of RAM and a 16 GB SD card for local storage. Each Raspberry Pi 4B will have the same input data (audio signal and watermark) and the same copy of the instructions script, but each node only runs a specific part of the script determined by the master Raspberry Pi of the cluster.

Five audio signals of different types and lengths from (https://www.looperman.com/loops) were used for the experiments as test audio signals (Table 2), and a binary sequence of ones and zeros was used as a watermark. The length of the watermark depends on the duration of the host audio signal. A single bit of the watermark is embedded in the host signal every 486 samples, covering the whole host signal.

**Table 2:** Information on the test audio signals.

Audio signal	Category	Duration (s)	Bits per sample	Sample rate (kHz)	Format
Classical_looperman-t-5360279-0234295	Classical	60	16	44.1	Wave
Rap_looperman-t-5460389-0236865	Rap	90	16	44.1	Wave
Jazz_looperman-t-5378703-0236794	Jazz	120	16	44.1	Wave
Pop_ooperman-t-0966004-0236621	Pop	150	16	44.1	Wave
Rock_looperman-t-3294503-0236832	Rock	180	16	44.1	Wave

The performance of the proposed audio watermarking system is compared with that of six notable audio watermarking systems, each chosen for specific reasons. These systems, namely FrCT-DTCWT^19^, DWT-DTMT^10^, DWT-DCT^9^, DCT-SVD^17^, DWT^3^, DCT^5, 16, 18^, and SVD^7^, were selected based on considerations such as the popularity and similarity of the employed transform domains for embedding and their proven track record of robustness against common signal processing manipulations. This comparison assesses the proposed system against these benchmarks in terms of payload capacity, imperceptibility, robustness of the watermark against common signal processing manipulations, and computational complexity.

### Payload capacity

The payload capacity determines the quantity of information that can be inserted into the host signal while maintaining imperceptibility. Let $B$ be the number of bits embedded into an audio signal of duration $d$ in seconds. Payload capacity is defined as follows:

23 $$P = \frac{B}{d}(bps)$$

The payload capacity $P$ is measured in the unit of bps (bits per second). According to the International Federation of the Phonographic Industry (IFPI)^44^, the payload capacity must be at least 20 bps for any audio watermarking system. Therefore, the payload capacity of the proposed system, shown in Table 3, is too high and very sufficient and verifies the IFPI condition, which is set at 20 bps.

**Table 3:** Payload capacity for different audio signals.

Audio signal	Length of watermark	Payload capacity
Classical_looperman-t-5360279-0234295	5438	90.7407 (bps)
Rap_looperman-t-5460389-0236865	8158	90.7407 (bps)
Jazz_looperman-t-5378703-0236794	10,945	91.4938 (bps)
Pop_ooperman-t-0966004-0236621	13,665	91.4938 (bps)
Rock_looperman-t-3294503-0236832	13,665	91.4938 (bps)

From the comparison results in Table 4, we can see that the proposed system can provide a high average payload (91.1926 bps), which is much higher than the 20 bps recommended by IFPI. The average payload of our system is higher than that of^9, 16–18^, but it is lower than that of other selected systems in this comparison. This can be justified by the fact that the payload of the proposed system was set to a sufficient and acceptable value in order to have superiority in terms of imperceptibility. Indeed, imperceptibility is the main requirement of any audio watermarking system; if the watermarked audio signal is not of good quality, it will not be accepted either by the industry or by the users.

**Table 4:** Comparison with six audio systems cited in the literature in terms of payload capacity.

Audio watermarking system	Average payload capacity (bps)
Proposed	91.1926
^19^	496.48
^10^	541.10
^9^	40.27
^3^	102.40
^5^	450.00
^7^	172.39
^16^	64.00
^17^	64.00
^18^	64.50

### Imperceptibility

For measuring the imperceptibility of the watermarked audio signals, the signal-to-noise ratio (SNR)^9^ is adopted to evaluate the quality of the watermarked audio signal by measuring the objective similarity between the original host signal $S = \{ s(i), \, 0 \le i < L\}$ and the watermarked one $\overline{S} = \{ \overline{s(i)} , \, 0 \le i < L\}$. A larger value of SNR indicates that the watermarked audio signal closely resembles to the original audio signal, which means that the watermark is more imperceptible. The SNR is defined and calculated as follows:

24 $${\text{SNR}}(S,\overline{S} ) = 10\log \left( {\frac{{\sum\limits_{i = 0}^{L - 1} {s^{2} (i)} }}{{\sum\limits_{i = 0}^{L - 1} {\left[ {s(i) - \overline{s(i)} } \right]^{2} } }}} \right)$$

According to the IFPI^44^, the SNR must be at least greater than 20 dB to have an imperceptible watermarked audio signal.

**Figure 8:**
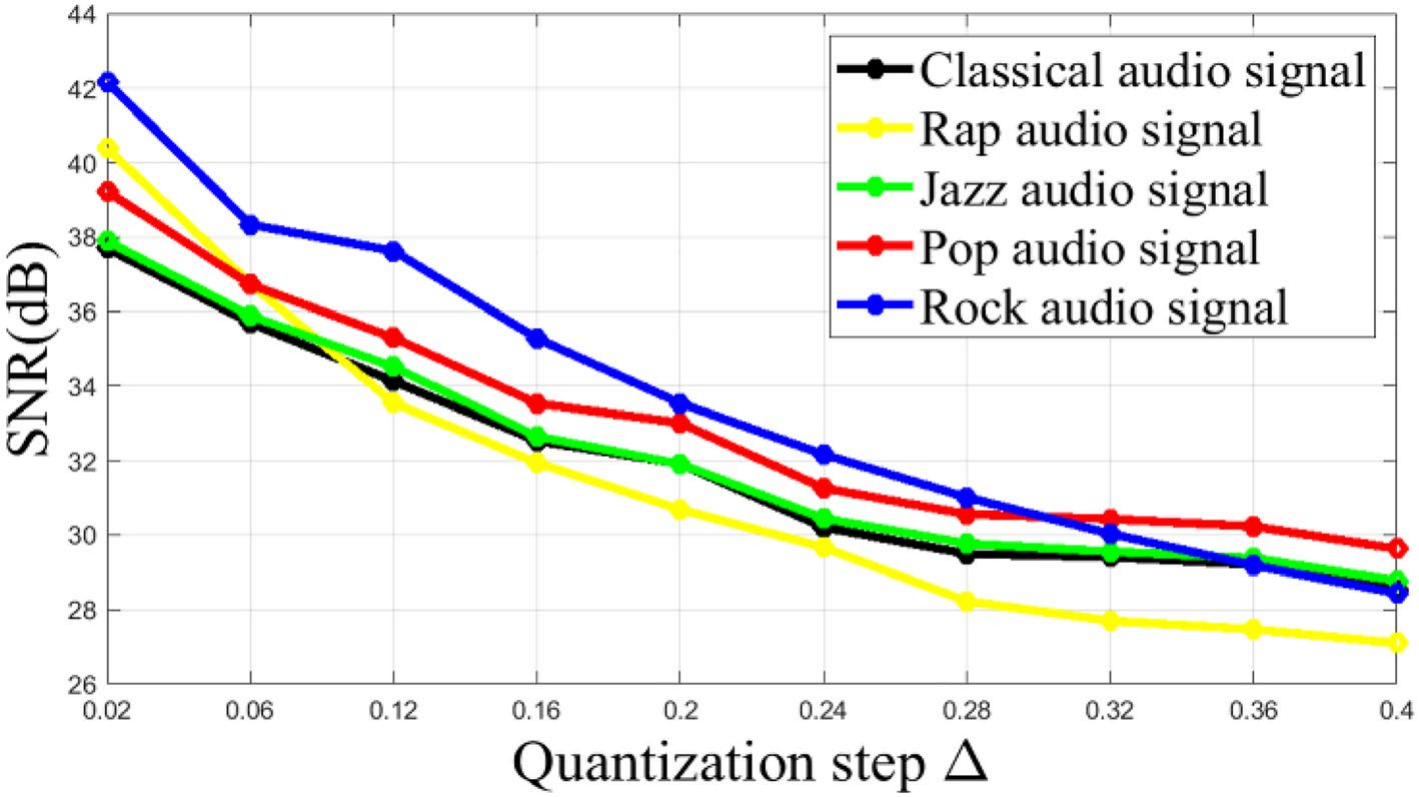
Imperceptibility for different watermarked audio signals with respect to quantization step Δ.

In our system, we embedded the watermark bits by quantizing the energies of FrCT coefficients. In general, in quantization-based audio watermarking systems, imperceptibility and robustness are influenced by the value of the quantization step Δ. A larger quantization step will result in a lower quality of the watermarked audio, while a smaller quantization step will influence the robustness of the watermark. In order to obtain the appropriate value of Δ, experiments were performed for different host audio signals. The binary watermark is embedded in the host audio signals with different quantization steps Δ. For each quantization step Δ, the SNR values are calculated and then plotted against Δ in Fig. 8. As expected, the SNRs decrease with increasing Δ. This is because the energies of the FrCT coefficients (where the watermark bits are embedded) are far from their original values, and thus there are distortions in the original audio signals. This figure also shows that the step Δ = 0.2 gives an SNR greater than 30 dB for different signals, which largely ensures the IFPI recommendation. Thus, this step value will be used in the following experiments.

**Figure 9:**
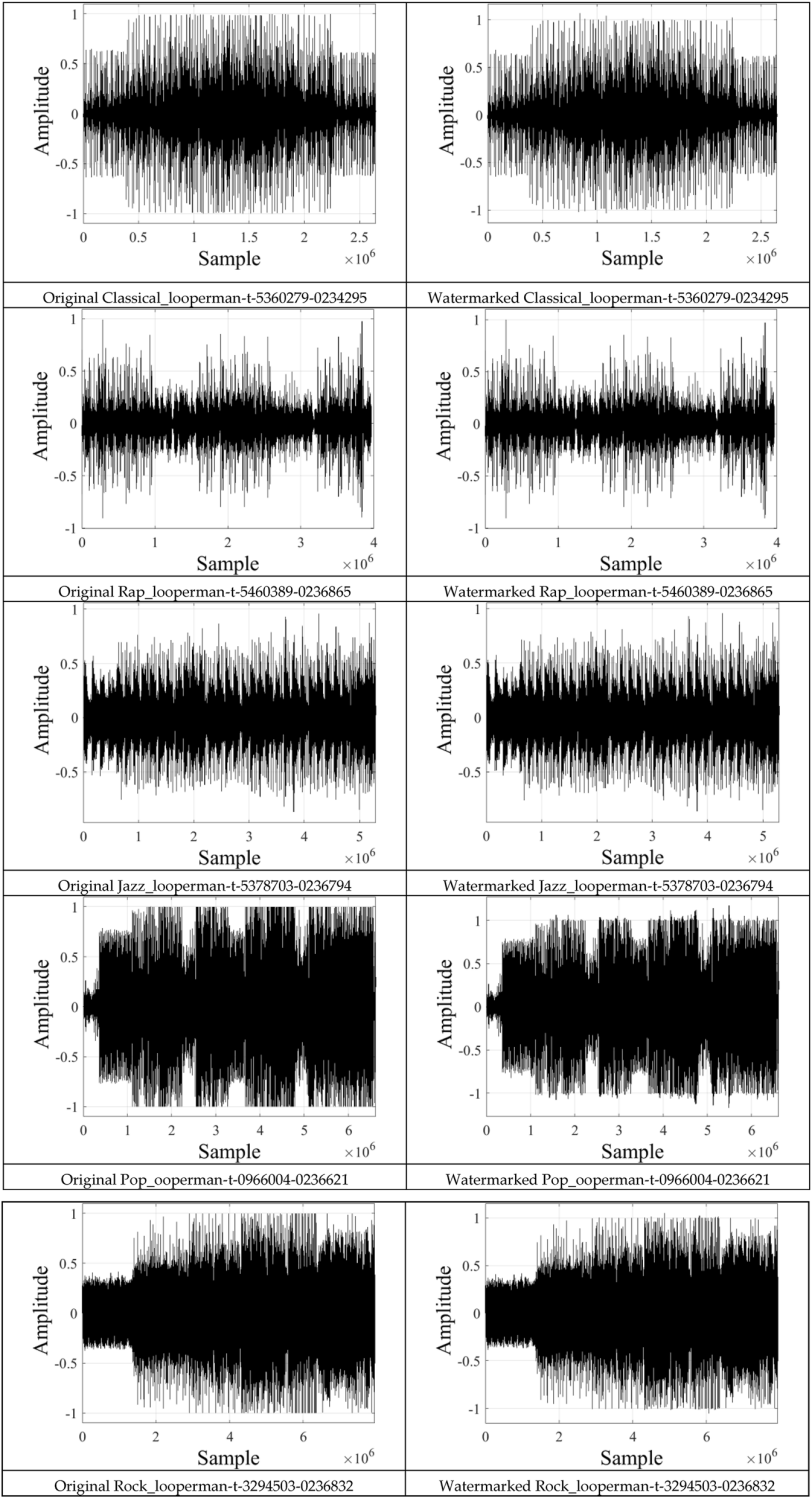
The original and watermarked audio signals with Δ = 0.2.

Figure 9 shows the original audio signals and the watermarked versions using Δ = 0.2, and the corresponding SNR values are listed in Table 5. These results clearly show that the proposed system satisfies the requirements of the IFPI with an SNR greater than 20 dB for different audio signals, and it can be increased up to 33.5 dB depending on the type of the host signal.

**Table 5:** SNR for different watermarked audio signals.

Audio signal	SNR (dB)
Classical_looperman-t-5360279-0234295	31.9157
Rap_looperman-t-5460389-0236865	30.6872
Jazz_looperman-t-5378703-0236794	31.9098
Pop_ooperman-t-0966004-0236621	33.0123
Rock_looperman-t-3294503-0236832	33.5415

The comparison results presented in Table 6 clearly show that the proposed system can achieve high imperceptibility (32.21 dB), which is much higher than the 20 dB recommended by IFPI. The average imperceptibility of our system is higher than that of most other systems selected for comparison. Note that the average imperceptibility of our system is lower than that of^5^ because we chose a relatively large quantization step in order to have good robustness. The advantage of this choice will be clearly demonstrated in the next section.

**Table 6:** Comparison with six audio systems cited in the literature in terms of imperceptibility.

Watermarking method	Average SNR (dB)
Proposed	32.2133
^19^	31.4936
^10^	29.1370
^9^	31.0786
^3^	22.46
^5^	35.3644
^7^	30.30
^16^	26.86
^17^	25.26
^18^	26.50

### Robustness against common signal processing manipulations

Watermarked audio signals can be frequently subjected to common signal processing manipulations. These manipulations can modify the frequency content and dynamics of the host audio signal and, as a result, deform the embedded watermark. In addition, third parties may attempt to modify the watermarked audio signal to prevent extraction of the embedded watermark.

To evaluate the robustness of the watermark against different common signal processing manipulations, the Bit Error Rate (BER)^12^ is used as an objective criterion in this paper. Mathematically, BER is defined as

25 $${\text{BER}} = \frac{{\text{Number of erroneously extracted bits}}}{{\text{Watermark length}}} \times 100\%$$

BER measures the similarity between the original watermark and the extracted one. BER is a number in the range [0, 1]. If BER is equal to 0, then the extracted watermark is exactly the same as the original one. If it is equal to 1, then the extracted watermark is very different from the original one, i.e., the extraction process has failed.

The robustness of the proposed watermarking system is evaluated against common signal processing manipulations and attacks. The robustness results of the proposed system are as follows:

#### Robustness without signal processing manipulations

The present test is performed to verify the effectiveness of the proposed system in recovering the watermark from watermarked audio signals without any applied manipulation. The extraction results for different audio signals (Table 2) are presented in Table 7. This table shows that the BER values are zeros for all audio signals, which clearly indicates the robustness of the proposed system in the absence of any possible manipulation. However, there are still many tests to be performed to validate the robustness of our system against common signal processing manipulations and attacks.

**Table 7:** Robustness results without applying signal processing manipulations.

	Classical	Rap	Jazz	Pop	Rock
BER	0	0	0	0	0

#### Robustness to AWGN

When transmitting watermarked audio signals to a radio station via a communication channel, these signals may be affected by noise. Therefore, it is necessary to test the robustness of the proposed system in noisy environments. In this context, the Additive white Gaussian noise (AWGN) is applied to the watermarked audio signals with SNR equal to 30 dB, 20 dB, and 18 dB. Then, the extraction process is applied to recover the embedded watermark from the noisy watermarked signals. The extraction results for the five audio signals are presented in Table 8. These results indicate that the proposed method is able to extract the watermark perfectly even with AWGN addition, and the BER values remain zero for different watermarked signals. Therefore, the proposed method is effectively resistant to noise addition.

**Table 8:** Robustness results (BER) in the case of noise addition.

Manipulation	Classical	Rap	Jazz	Pop	Rock
AWGN (30 dB)	0	0	0	0	0
AWGN (20 dB)	0	0	0	0	0
AWGN (18 dB)	0	0	0	0	0

#### Robustness to resampling and requantizing

Resampling and requantizing are common signal processing manipulations that change the format of the watermarked signals. During the experiment, the watermarked signals were firstly down-sampled to 8000 Hz, 11,025 Hz, and 22,050 Hz, and then up-sampled back to 44,100 Hz. Secondly, the watermarked signals were quantized to 24 bits/sample, 8 bits/sample, and then back to 16bits/sample. The extraction results of these manipulation attacks are presented in Table 9. We can observe from this table that the BERs are zeros for different watermarked signals, which proves that the proposed method can effectively resist resampling and requantizing.

**Table 9:** Robustness results (BER) to resampling and requantizing.

Manipulation	Classical	Rap	Jazz	Pop	Rock
Resampling (22,050 Hz)	0	0	0	0	0
Resampling (11,025 Hz)	0	0	0	0	0
Resampling (8000 Hz)	0	0	0	0	0
Requantization (16–8–16 bits)	0	0	0	0	0
Requantization (16–24–16 bits)	0	0	0	0	0

#### Robustness to signal filtering

Filters are often used in signal processing to cut or remove certain sub-bands of the audio spectrum. For this, we evaluate the robustness of the proposed system against signal filtering. The watermarked audio signals are filtered by low-pass filtering with a cutoff frequency of 4 kHz and 500 Hz, respectively, and by high-pass filtering with a cutoff frequency of 200 Hz. The results of the filtering manipulations (Table 10) show that the BER values are lower than 1.60% for different filtered watermarked signals, which indicates that the proposed algorithm has strong robustness towards the filtering manipulations.

**Table 10:** Robustness results (BER) to signal filtering.

Manipulation	Classical	Rap	Jazz	Pop	Rock
Low-pass filtering (4 kHz)	0	0	0	0	0
Low-pass filtering (500 Hz)	1.5994	1.5922	1.5814	1.5650	1.5647
High-pass filtering (200 Hz)	1.3357	1.3363	1.3503	1.3398	1.3592

#### Robustness to echo addition

In this experiment, the robustness of echo addition is tested. We added to the watermarked signals an echo signal with a delay of 50 ms and a decay of 5% and an echo signal with a delay of 300 ms and a decay of 40%. Table 11 presents the BERs after adding these modifications. The extraction results show that the BERs are zeros for all watermarked signals, which indicates that the proposed algorithm has strong robustness against echo addition.

**Table 11:** Robustness results (BER) to echo addition.

Manipulation	Classical	Rap	Jazz	Pop	Rock
Echo addition (50 ms, 5%)	0	0	0	0	0
Echo addition (300 ms, 40%)	0	0	0	0	0

#### Robustness to MP3 compression

Signal compression is often applied to audio signals during processing to reduce the size of audio files. We test, in Table 12, the robustness of the proposed system when the watermarked signal format is changed from WAVE to MP3 and back to WAVE by applying MPEG-1 Layer 3 compression with 128 kbps, 112 kbps, 64 kbps, and 32 kbps. As seen from this table, the proposed system still has very low BERs when MP3 (32 kbps) is applied, which are less than 11.40%. That means that the proposed system provides good performance under MP3 compression manipulations.

**Table 12:** Robustness results (BER) to MP3 compression.

Manipulation	Classical	Rap	Jazz	Pop	Rock
MP3 (128 kbps)	0	0	0	0	0
MP3 (112 kbps)	0	0	0	0	0
MP3 (64 kbps)	9.0872	9.1195	8.0508	8.4168	7.8226
MP3 (32 kbps)	11.0870	10.2092	10.4080	11.3230	10.6726

#### Robustness to amplitude scaling

The robustness of the proposed system is also tested against amplitude scaling manipulation. We scaled the amplitude of the watermarked audio signals with factors of 1.2, 1.1, 0.9, and 0.8, and then the extraction process was applied to recover the embedded watermark. Table 13 presents the extraction results in terms of BER. These results show that the BERs are zeros, which indicates that the proposed system has strong robustness against amplitude scaling manipulation.

**Table 13:** Robustness results (BER) in the case of existing amplitude scaling.

Manipulation	Classical	Rap	Jazz	Pop	Rock
Amplitude scaling (1.2)	0	0	0	0	0
Amplitude scaling (1.1)	0	0	0	0	0
Amplitude scaling (0.9)	0	0	0	0	0
Amplitude scaling (0.8)	0	0	0	0	0

#### Robustness to cropping

In this experiment, the robustness against cropping manipulation is tested. Cropping is a manipulation frequently applied by third parties to modify watermarked signals to distort the embedded watermark. In this test, 10%, 20%, 30%, and 40% samples of the watermarked signals are randomly replaced by zeros. The results for the watermarked signal of classical, rap, jazz, pop, and rock are given in Table 14, indicating that the proposed system has strong robustness against cropping manipulation where the BER does not exceed 1.2% for random cropping (40%).

**Table 14:** Robustness results (BER) in the case of existing cropping.

Manipulation	Classical	Rap	Jazz	Pop	Rock
Random cropping (10%)	0	0	0	0	0
Random cropping (20%)	0	0	0	0	0
Random cropping (30%)	0	0	0	0	0
Random cropping (40%)	1.1785	1.1777	1.1004	1.1467	1.0438

#### Robustness to shifting

Shifting is another very sophisticated manipulation that can be used to distort the embedded watermark by shifting the watermarked audio signal by a specified number of samples to the right or to the left. In this test, the performance of the proposed system is tested under image translation signal shifting: the watermarked audio signal is shifted to the right by 5, 10, 20, 50, 100, and 150 samples, and then the extraction process is applied to recover the embedded watermark. Table 15 shows that the proposed system achieves superb robustness against shifting manipulation when 5, 10, 20, and 50 samples are shifted and acceptable robustness when 100 and 150 samples are shifted with BERs less than 0.152 (15.2%), which is expected because the DTCWT transform adopted by the proposed system ensures shifting invariance.

**Table 15:** Robustness results (BER) in the case of existing shifting.

Manipulation	Classical	Rap	Jazz	Pop	Rock
Shifting (5 samples)	0.0341	0.0396	0.0375	0.0272	0.0386
Shifting (10 samples)	0.0793	0.0850	0.0759	0.0942	0.0869
Shifting (20 samples)	0.8171	0.6985	0.8313	0.7244	0.7185
Shifting (50 samples)	2.2274	2.2035	2.2399	2.2358	2.2417
Shifting (100 samples)	8.6434	8.4967	8.5547	8.5606	8.6416
Shifting (150 samples)	15.0390	15.1003	15.1723	15.1292	15.1588

#### Robustness to TSM

Time Scale Modification (TSM) is a digital signal processing technique employed to either accelerate or decelerate the playback speed of an audio signal without altering its pitch. TSM can be utilized for various purposes, such as adjusting the duration of music recordings to ensure synchronous playback or synchronizing an audio signal with a given video clip. In this test, we evaluate the performance of the proposed system when subjected to TSM. TSM is applied to watermarked audio with varying degrees of modification ranging from − 5 to + 5%. Subsequently, the extraction process is executed to recover the embedded watermark. The results, presented in Table 16, showcase the extraction performance in terms of BER. Notably, these results indicate that the proposed system maintains consistently low BER values, all of which are less than 11%, even when subjected to TSM with different degrees of modification. This test underscores the robust performance of the proposed system when subjected to TSM manipulations.

**Table 16:** Robustness results (BER) to TSM.

Manipulation	Classical	Rap	Jazz	Pop	Rock
TSM (+ 1)	5.6129	5.0856	5.5747	5.3971	5.7445
TSM (− 1)	6.4553	6.4992	5.9417	6.1319	6.3586
TSM (+ 5)	9.9908	9.9700	9.4926	9.6817	9.7023
TSM (− 5)	10.1900	10.2025	10.0320	9.7840	10.1078

The robustness of the proposed audio watermarking system is evaluated through a comparative analysis with nine state-of-the-art audio watermarking systems. The results of this comparison are summarized in Table 17. It is clear that the proposed audio system demonstrates greater robustness compared to audio systems in Refs.^3, 5, 7, 9, 10, 16–19^, overall, and is only slightly less effective than^3^ in terms of MP3 compression resistance, as well as^17^ in terms of TSM resistance. Our method, along with methods^16, 17, 19^, stands out for its resistance to shift attacks. This resistance can be attributed to the use of the DTCWT transform in our method and in method^19^, providing an approximate shift invariance. Methods^16, 17^ also achieve significant resistance through a synchronization mechanism.

**Table 17:** Comparison with six audio systems cited in the literature in terms of robustness.

Manipulation	Proposed	^19^	^10^	^9^	^3^	^5^	^7^	^16^	^17^	^18^
No modification	0	0	0	0	0	0	0	0	0	0
AWGN (20 dB)	0	0	0	0	0	0.24	4.25	2.42	0.07	4.46
AWGN (15 dB)	0.2458	0.23	1.43	1.53	3.56	6.40	11.32	10.54	9.24	12.78
Resampling (22,050 Hz)	0	0	0	3.90	0	0.10	0	0.32	0.70	0.790
Resampling (11,025 Hz)	0	0	0	6.81	0	4.02	0	6.64	7.82	8.74
Resampling (8000 Hz)	0	0	0	11.02	0	9.33	4.30	12.81	13.52	13.46
Requantization (16–8–16 bits)	0	0	0	1.81	0	0.10	0.85	0	0	0.36
Requantization (16–24–16 bits)	0	0	0	0	1.35	0	0	0	0	1.45
Echo addition (300 ms, 40%)	0	0	0	0	7.74	8.95	6.27	9.46	8.43	9.87
Random cropping (10%)	0	0	0	0	0.37	0	12.58	0.61	0.63	1.05
Random cropping (20%)	0	0	0	0	0.68	0	13.26	3.97	4.64	3.87
Random cropping (30%)	0	0	0	0	1.59	0.67	17.37	8.56	8.04	7.21
Random cropping (40%)	1.13	1.38	0.35	1.25	14.99	0.92	27.65	16.10	16.57	17.54
Low-pass filtering (4 kHz)	0	0	0.51	0.54	0.91	2.32	9.35	0.15	25.64	18.54
Low-pass filtering (500 Hz)	1.58	1.76	1.09	11.83	2.03	21.84	20.17	22.49	36.74	36.48
High-pass filtering (200 Hz)	1.34	1.35	2.84	4.78	2.96	26.05	25.80	22.65	21.97	20.87
Amplitude scaling (0.7)	0	0	0	0	22.04	25.25	0.49	0	0.96	0.24
MP3 (128 kbps)	0	0	0	0	0	0.03	5.30	0.76	0.79	0.68
MP3 (112 kbps)	0	0	0	0	0	3.44	9.36	0.94	1.02	0.85
MP3 (64 kbps)	8.45	8.08	7.31	24.05	0	26.07	22.11	10.10	11.28	9.47
MP3 (32 kbps)	10.74	10.47	25.77	31.80	0	32.30	39.90	28.94	29.45	28.04
Shifting (5 samples)	0.03	0.02	38.40	38.38	36.30	42.28	39.55	3.04	0.02	39.78
Shifting (10 samples)	0.08	0.037	40.17	42.96	39.62	47.40	45.03	4.10	0.13	41.97
Shifting (20 samples)	0.76	0.78	46.58	47.63	46.11	50.03	44.15	6.84	0.89	42.79
Shifting (50 samples)	2.23	2.37	41.60	46.26	47.67	51.41	47.96	8.27	2.46	45.10
Shifting (100 samples)	8.58	8.63	48.36	47.29	45.47	54.57	46.24	17.58	3.94	45.34
Shifting (150 samples)	15.12	15.31	50.46	49.56	50.18	51.06	50.39	21.05	10.78	48.46
TSM (− 1%)	6.28	7.53	14.46	11.96	6.89	12.87	26.40	6.17	1.84	12.54
TSM (− 5%)	5.48	7.01	13.96	11.11	6.65	13.14	25.39	5.84	1.47	11.30
TSM (− 5%)	10.06	15.61	19.79	17.12	14.14	26.80	37.82	12.07	3.34	14.46
TSM (+ 5%)	9.77	15.53	19.51	16.09	14.29	26.75	37.35	11.46	2.69	14.07

### Time complexity analysis

**Figure 10:**
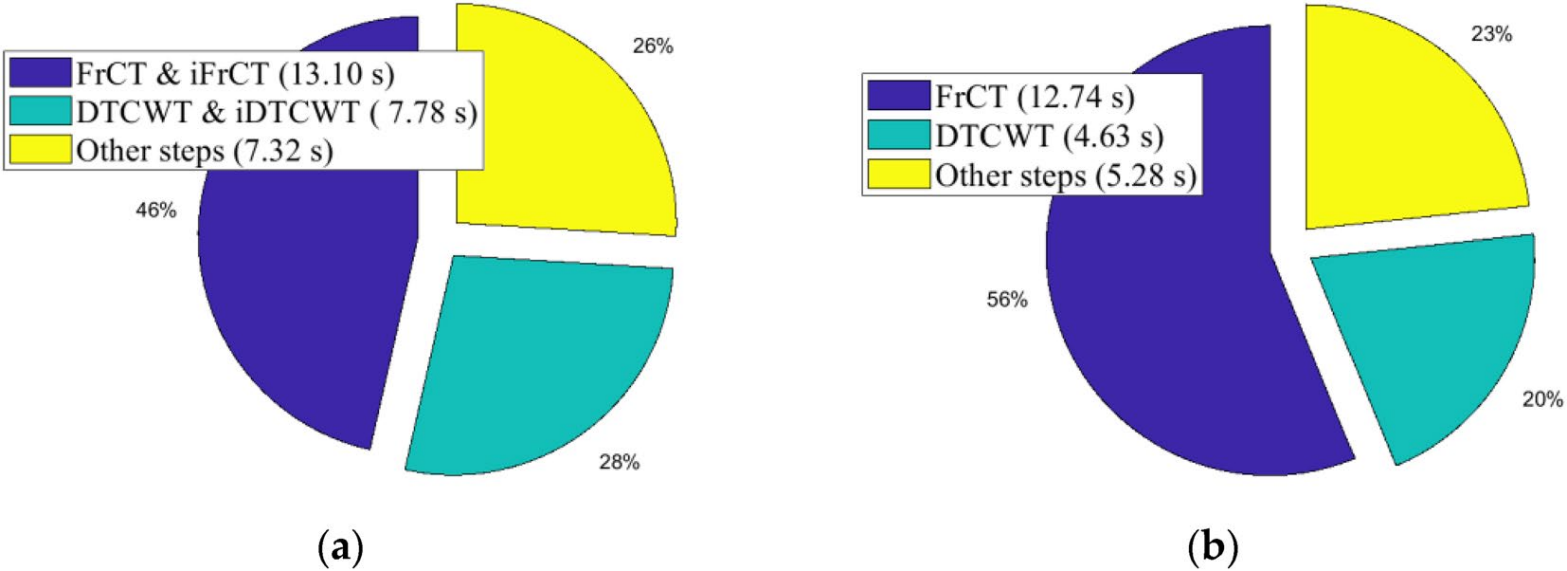
The sequential execution time in the proposed watermarking system for the classical audio signal of duration of 60 s: (**a**) embedding process, (**b**) extraction process.

Transform domain watermarking systems are more robust than those implemented in the time domain. However, the major disadvantage of transform-domain watermarking systems is that they are time-consuming, especially for audio signals of high duration. Table 18 shows the elapsed execution time of the proposed audio watermarking system implemented on the Raspberry Pi using the sequential approach. This test was performed using different audio signals (Classical, Pop, Jazz, Rap, Rock) with durations ranging from 60 to 180 s. As shown in this table, the execution time of the embedding and extraction processes is very high, and it increases with increasing signal duration. Figure 10 shows the time required for different steps of the proposed system using the “Classical” audio signal of 60 s. As shown in this figure, the most computationally intensive steps are the computation of the transforms FrCT and DTCWT and their inverse transforms iFrCT and iDTCWT. This leads to slow embedding and extraction processes.

**Table 18:** Sequential execution times of the proposed audio watermarking implemented on a single Raspberry Pi 4B cluster.

Audio signal with its duration	Execution time (in seconds)	
	Embedding process	Extraction process
Classical (60 s)	28.2114	22.6615
Rap (90 s)	52.5263	47.71793
Jazz (120 s)	92.7756	86.9927
Pop (150 s)	120.2145	115.9767
Rock (180 s)	181.1722	173.6886

**Figure 11:**
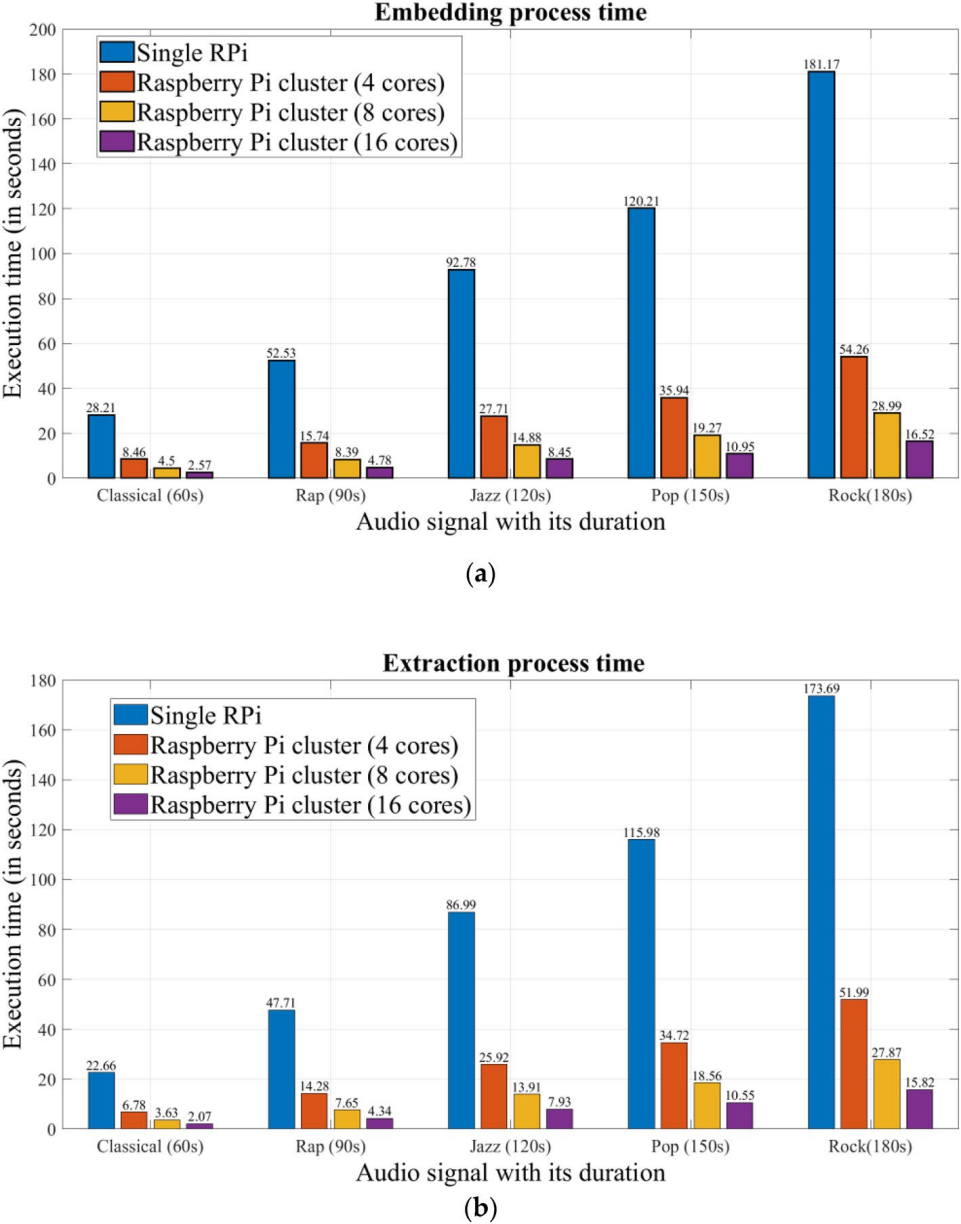
Execution time (in seconds) of the parallel audio watermarking system implemented on our Raspberry Pi cluster: (**a**) Embedding process, (**b**) Extraction process.

As highlighted in Section "Proposed parallel audio watermarking system", both the embedding and extraction processes can be parallelized using the MPI library on the Raspberry Pi cluster to accelerate the execution time of the proposed audio watermarking system. Each Raspberry Pi 4B in our cluster (Section "Raspberry Pi cluster") has the same input data and the same copy of the script, but each node executes only a specific part of the script determined by the master Raspberry Pi of the cluster. All raspberry pi's in the cluster are independent of each other, and communications (sending and receiving data) between the master node and the other nodes are ensured using MPI. Figure 11 presents the execution times required by the proposed parallel audio watermarking system implemented on our Raspberry Pi cluster. This test was performed on different numbers of cluster cores and different audio signals. This figure shows the superiority of the proposed parallel system implemented on the cluster compared to the sequential system implemented on a single Raspberry Pi of the cluster. The efficiency of the proposed approach increases with an increasing number of cores used in the cluster.

In order to measure the effectiveness of the proposed approach, we use the Execution Time Improvement Ratio (ETIR)^45^, which represents the comparison ratio between the execution time of the sequential watermarking system and the execution time of the parallel watermarking system implemented on the Raspberry Pi cluster. ETIR is defined as follows:

26 $$ETIR = \frac{{T_{Sequentiel} - T_{Parallel} }}{{T_{Sequentiel} }} \times 100$$

The obtained ETIR values of the proposed parallel system on different cores of the Raspberry Pi cluster are presented in Table 19. This table shows that the proposed parallel system is largely fast compared to the sequential system, with time improvements of about 70%, 80%, and 90% using 4, 8, and 16 cores of the cluster, respectively, which proves the effectiveness of the proposed method in terms of speed.

**Table 19:** Temporal improvement (ETIR) using the proposed parallel audio watermarking system implemented on our raspberry pi cluster for various audio signals.

Audio signal with its duration	RPi cluster (4 cores)		RPi cluster (8 cores)		RPi cluster (16 cores)	
	Embedding process	Extraction process	Embedding process	Extraction process	Embedding process	Extraction process
Classical (60 s)	70.02	70.06	84.02	83.99	90.88	90.88
Rap (90 s)	70.04	70.07	84.02	83.96	90.90	90.90
Jazz (120 s)	70.13	70.21	83.96	84.01	90.89	90.88
Pop (150 s)	70.10	70.07	83.97	83.99	90.89	90.90
Rock (180 s)	70.05	70.07	84.00	83.96	90.88	90.89

The comparison results presented in Table 20 clearly demonstrate the substantial performance advantage of our proposed parallel system when it is implemented on our multi-core Raspberry Pi cluster in comparison to its sequential counterparts executed on different computing platforms, including the AMD Ryzen 5 PC and the Intel Core i3 PC. For instance, for a 60-s audio signal with a 5438-bit watermark, our proposed system exhibits significantly reduced processing times. Specifically, the proposed system achieves execution times of just 7.6210 s, 4.0680 s, and 2.3197 s when deployed on 4, 8, and 16 cores of the Raspberry Pi cluster, respectively. In contrast, the same computation necessitates 49.2675 s on the AMD Ryzen 5 PC and 19.6285 s on the Intel Core i3 PC. These results unequivocally underscore the marked performance advantage of our parallel approach when implemented on the multi-cores of the Raspberry Pi cluster. Furthermore, this performance improvement is consistently observed when our parallel approach is applied to other audio watermarking schemes, such as those referenced in Refs.^9, 10, 19^, utilizing the Raspberry Pi parallel system as detailed in the manuscript (refer to Table 20). These outcomes consistently demonstrate the effectiveness and efficiency of our proposed parallel approach across a spectrum of audio watermarking schemes.

**Table 20:** Average execution times for 60 s audio signal with 5438-bit watermark in proposed parallel watermarking and existing systems.

Implementation environment	Audio watermarking system			
	Proposed	^10^	^9^	^19^
PC Intel core i3 2.40 GHz, 6 GB RAM,	49.2675	58.4176	37.9481	71.3133
PC AMD Ryzen 5 2.30 GHz, 8 GB RAM	19.6285	23.5018	10.8438	29.1655
Raspberry Pi cluster (4 cores)	7.6210	7.8491	5.9706	8.4218
Raspberry Pi cluster (8 cores)	4.0680	4.1787	3.7922	4.5469
Raspberry Pi cluster (16 cores)	2.3197	2.6324	1.9572	2.9649

## Conclusion

Information security is becoming more and more essential with the increase in data exchange on the internet. The security of multimedia data, such as digital audio, can be practically achieved by digital watermarking. However, traditional sequential watermarking systems are becoming too inefficient for real-time applications. A parallel watermarking system for audio signals that can be executed in a few seconds is presented in this paper. The repetitive and intensive steps in the audio watermarking system have been parallelized in order to execute them simultaneously on a set of physical cores in a cluster. The cluster of four Raspberry Pis thus adopted is characterized by its easy portability, low power consumption, portability, and low cost, which is very important for watermarking-based smart city applications. The experimental results showed the preference of our parallel system not only in terms of computation time but also in terms of the imperceptibility and robustness of the watermark against common signal processing manipulations.

## Data Availability

All data are available upon request from the first author (Mohamed Yamni, mohamed.yamni@usmba.ac.ma).
